# Factors Associated with the Establishment of New Occupational Therapist Positions in Norwegian Municipalities after the Coordination Reform

**DOI:** 10.1177/1178632921994908

**Published:** 2021-03-16

**Authors:** Ruca Maass, Tore Bonsaksen, Astrid Gramstad, Unni Sveen, Linda Stigen, Cathrine Arntzen, Sissel Horghagen

**Affiliations:** 1 Departement of Nevromedicine and Movement Science, Faculty of Medicine and Health Science, Norwegian University of Science and Technology, Norway; 2 Departement of Health and Nursing Sciences, Faculty of Social and Health Sciences, Inland Norway University of Applied Science, Elverum, Norway; 3 Departement for Health and Care Sciences, The Arctic University of Norway, Norway; 4 Center for Care Research, North; 5 Departement for Occupational Therapy Prosthetics and Orthotics, Faculty of Health Sciences, Oslo Metropolitan University, Norway; 6 Departement of Health Sciences, Gjøvik, Faculty for Medicine and Health Sciences, Norwegian University of Science and Technology, Norway

**Keywords:** Community-based occupational therapy, primary care, access to health services, health equity

## Abstract

Community-based occupational therapy is an increasingly important domain of work for occupational therapists. In Norway, this has been emphasized by the Coordination reform (2012), which assigned municipalities increased responsibility to protect and promote the health of their inhabitants. However, even if approximately 400 positions have been established between 2012 and 2017, little is known whether they have contributed to increased and/or more equal coverage across municipalities. To explore this matter, survey data was gathered among members of the Norwegian Occupational Therapy Association during 2017. Data was analyzed statistically (descriptive, comparative and associative) with SPSS 25. Results suggest large regional variations in the establishment of new positions. Moreover, most new positions were established in medium-sized municipalities that already had (an) occupational therapist(s) in the community. Number of prior positions, as well as being in the process of merging with another municipality were the only significant predictors for the establishment of new positions during regression analysis. Findings suggest that no levelling-out of geographical distributions of OT-coverage has occurred, even if new positions might have contributed to level-out workload (number-of-patients-per-therapist). Further, we discuss implications of our findings for policy-making and recruitment of Occupational Therapists for rural positions.

## Introduction

Occupational Therapy (OT) was established as a municipal service in 1987, at first in 11% of Norwegian municipalities.^1^ Since then, occupational therapy has become an increasingly central part of community-based (municipal) health services, in Norway as well as in other parts of the world.^2-8^ However, coverage of occupational therapy differs substantially between municipalities, contributing to differences in available health services and thereby, in its utmost consequence, to social inequality in health.

Community-based occupational therapy contributes with knowledge on, and approaches aiming at, participation and health in everyday-life for a wide range of user groups. It is a central part of multi-professional rehabilitation approaches in community-based teams, while also applying approaches aiming at health promotion and prevention.^7^ Assistive and welfare technology is an important part of community-based OT, which accounted for over 51% of occupational therapists’ working time in a recent Norwegian study.^8^ A qualitative study finds that providing assistive technology also emerged as the main role for OTs working in the community.^9^ However, some of the participants experienced that other professions underestimated or under-communicated the professional competency needed to assess needs and adapt assistive technology. Accordingly, they evaluated the role as provider of assistive technology as less attractive and less socially valued than other roles,^9^ and occupational therapists (OTs) using most of their working time on assistive technology experienced less influence over decisions in their municipality.^10^

Other typical tasks for community-based OTs include the enablement of independent living for various user-groups, including patients with neurological conditions like dementia or stroke,^11,12^ the prevention of ill health or loss of activity, for example through home-visits for preventing falls,^12,13^ as well as reablement. Reablement has emerged as a central strategy which aims at creating sustainable change and increased participation in everyday-life.^14^ Reablement includes increased security and participation in meaningful activity by focusing on assets, skills and resources.^15^ A crucial part of these approaches is working with the social network of the client, including family and adult children.^15,16^ Last, large proportions of Norwegian occupational therapists (more than 40%) are involved in, or lead, research or innovative projects in their municipality. Of those who were not involved in any project, almost 70% expressed the wish to do so.^17^

Community-based OT has received increased attention in recent years. Community- based services undergo profound changes in general, and occupational therapy is in policy documents described as an important part of service delivery in the Nordic countries, as well as in an international perspective.^3-6^ In Norway, community-based OT was put on the agenda in 2012, when the so-called ‘Coordination Reform’ was implemented. The reform implied a shift in the role of municipalities regarding health care provision: the reform aimed to offer adequate services at the right time and place, and move the provision of services closer to where people ‘live, work and play’ and the settings for everyday-life.^18^ Hence, the municipality is described as the main provider of health services. Community-based health services have been given extended responsibilities to monitor, protect and promote the health of inhabitants. Community-based OT came into focus, as approaches like reablement, welfare technology and participation in various activities emerged as valid strategies to reach these goals.^8,9,19-21^

The Coordination Reform emphasized the municipalities’ role in respect to health and health equity, as also expressed in the sustainable development goals ‘good health’ and ‘reduced inequalities’.^22^ Access to adequate health services has been described as an important factor in health equity.^23^ Despite low social inequality, geographic variations in longevity and health are quite pronounced in Norway.^24^

The Norwegian Occupational Therapy Association has emphasized the need for better, and more consistent coverage of OT within municipalities.^19,25^ Community-based OT has, until recently, not been a mandatory health service: the decision to employ occupational therapists was up to each municipality. Accordingly, differences in municipal policy regarding OT contributed to different access to OT-services between inhabitants of different municipalities, contributing to inequality in access to health services. At the end of 2017, 406 of 475 (84%) municipalities offered OT-services to their inhabitants.^10^ In 2018, 2338 community-based OT-positions were registered,^1^ which was an increase of 400 positions during 4 years.^26^ However, whether this increase describes the establishment of OT services in new municipalities, or if municipalities with already existing OT-units increased their capacity, is unknown. Some evidence suggests that more knowledge about OT might contribute to increased demand for OT.^27-31^ It might therefore be reasonable to expect municipalities that already have occupational therapists to seek to increase their stock, whereas municipalities without occupational therapists (OTs) may be less inclined to establish novel positions.

However, differences might occur both in terms of the presence or absence of OT-services in the community and in respect to each occupational therapists’ caseload.^9,19^ According to Arntzen and colleagues^9^ did rural OTs experience more work-load, and less time per client, than their urban counterparts. Likewise, quality of services can differ between municipalities, indicating a rural-urban divide: OTs in rural positions experienced their work as more related to ‘fire-extinguishing’ and providing assistive technology, while urban OTs could make more use of their experience and competencies, and assumed more valued work roles such as ‘all-rounder’ and ‘innovator’.^9^ Urban OTs might also be able to achieve higher degrees of specialisation, partly due to a different organisation of health and OT-services, like the existence of multi-disciplinary specialized teams (working specifically towards reablement or health-promotion).^31^

Thus, social inequality in access to community-based OT can occur in respect to coverage absolutely (existing community-based OT or not) and relatively (client per OT), as well as in respect to quality (like access to specific competence and expertise).^9,32^ In combination with the Coordination Reform, emphasizing the importance of adequate local health services, this highlights the need for community-based occupational therapy across municipalities.

In 2014, Norwegian municipalities underwent another reform, which implied that 119 municipalities were planned to merge into 49 bigger entities. Whether, and how, this merge influenced the establishment of new OT-positions is not known: even if the merging of municipalities did not begin before 2015, its outlines and possible implications were visible already in 2012. It might be reasonable to expect that municipalities preparing for a merge established fewer new positions, either because they expected to be merged with another municipality already having OT-services, or because the anticipation of major changes prevented the establishment of new positions, and especially, introducing new professions.

Taken together, insecurity is linked to whether the newly established positions contributed to better coverage with community-based OT services, and if and how prospects of merging (implying a major restructuring) have influenced the establishment of new OT-positions. Accordingly, this study aims to explore contextual factors associated with the establishment of new OT-positions in conjunction with the implementation of the Coordination Act in 2012.

Possible links between the establishment of new OT-positions and size of the municipality (number of inhabitants), region, as well as the number of already existing OT positions, geographical organisation (with other health services or not) and OTs in leading positions are explored. Last, we want to examine whether, and how, the prospect of merging with other municipalities influenced the described relationships.

## Methods

Statistical data was gathered by survey, which was distributed to 1767 members of the Norwegian Occupational Therapy Association during May 2017. The association claims to organize 2/3 of all Norwegian Occupational Therapists.^33^ The survey contained a range of questions including personal background, as well as place, organisation of their workplace and daily work. The survey was returned by (*N* = 561), resulting in a response rate of 31%. The design of the study has been approved by the Norwegian Center for Research Data (project number 52827).

The dependent variable, *establishment of new OT-positions*, was assessed by a single item, asking OT in community service whether new positions had been established, with three possible answers; ‘yes’, ‘no’ and ‘I don’t know’.

Independent variables were included based on their expected relevance for the establishment of new OT positions:

*Size* of the municipality is described based on the number of inhabitants. Size is measured in 4 categories, <2000; 2000-19.999; 20.000-99.999; >100.000.

*Region* describes a geographical unit, as well as an organisational level within the Norwegian Health system. Health Regions have the responsibility to offer specialized health services to inhabitants. All 6 regions are included.

*Merging* is constructed out of 2 items, ‘already implemented’ or ‘decided upon’ a merge with another municipality. Both items are combined into a binary variable, with ‘1’ indicating the answer ‘yes’ to either question, while ‘0’ indicates ’no’ or ’I don’t know’.

Four variables describe existing OT-services, and how they are organized within the respective municipalities:

*Prior OT-positions* describes the number of already existing OT positions in the respective municipality/department/city-district. ‘Department’ does here refer to a specific field-of-responsibility within a geographical and organisational unit, for example, the ‘sykehjemsetaten’ (department of nursing homes) in Oslo, which is responsible for all elderly care homes in the Norwegian capital. ‘City-district’ describes an organisational level of community-based health services in bigger cities.

Next, *Co-placement* is a binary variable, describing whether OTs working in community-based services are physically located together with other occupational therapists or not.

Two included variables describe whether OTs hold leading positions, that are relevant for recruitment and establishment of new positions: *occupational therapist in leading position*, a categorical question whether the respondent has an occupational therapist as leader, and *respondent in leading position*, if the respondent herself has responsibility for human resources, recruitment and employment.

### Statistical analysis

Data were analyzed using SPSS 27. All included variables were described through proportions and numbers in total (for categorical variables) or by their mean and standard deviation (for scales). To gain first insights into structural and contextual differences, comparative analysis (applying Chi-square-tests for categorical, *t*-test for continuous variables) was conducted between participants answering ‘yes’, ‘no’ and missing cases. The latter was done to gain a clearer picture of factors that might influence knowledge about the establishment of new OT positions.

Missing cases/cases (answering ‘I don’t know’ on the dependent variable) were excluded from further analysis. Initial correlational analysis was carried out by estimating Pearson’s correlation coefficient. Variables significantly correlated with the dependent variable (the establishment of new positions) were included in further analysis. Thus, ‘respondent in a leading position’ was excluded from further analysis.

Then, a 2-step hierarchical regression analysis was conducted. All contextual variables significantly associated with the outcome variable in the bivariate analysis were entered in the first step. In the next step, we entered if the municipality had been, or was planned to be merged. This is done to both catch direct influences of ‘merging’ on the establishment of new positions, and simultaneously examine if and how this event impacted the influences of the stable, contextual variables entered in step 1.

## Results

Descriptive analysis (Table 1) revealed that most participants came from the Region Southeast (*n* = 162), followed by Vest (*n* = 107), while least participants were linked to Region North (*n* = 60) and South (*n* = 69). About 40% of all participants worked in medium-sized (2000-19.999) municipalities, while least worked in bigger cities (>20.000 inhabitants). The average number of OT-positions within the same unit was roughly 9.4; however, a standard deviation of almost 13 indicates major differences between municipalities. Two-thirds of all participants were co-placed with other OTs. However, less than a quarter reported having an occupational therapist in a leading position, and only 6,4% reported to be in a leading position themselves.

**Table 1.** Descriptive statistics.

		Whole sample (*n* = 561)	
		*N*	%
Region	North	60	10.7
	Middle	76	13.5
	West	107	19.1
	South	69	12.3
	East	87	15.5
	South-East	162	28.9
Size of municipality	<2.000	207	36.9
	2.000-19.999	235	41.9
	>20.000	119	21.2
New positions established within unit (% yes)		96	17.1
Co-placement with other OTs (% yes)		376	67
OT in leading position		128	22.8
Participant in leading position		36	6.4
Merged (% yes)		157	28
Number of OT’s in same unit (mean, std)		9.44 (12.9)	

A small proportion (3.4%) of participants reported working in a municipality that had already been merged, while about a quarter reported that future merging had been decided, but not implemented yet. About 17% of respondents (*N* = 96) reported that new OT-positions were established within their unit; while 42.8% (*n* = 240) answered ‘no’, and 40.1% (*n* = 225) answered ‘I don’t know’ to this question.

### Comparative analysis

Comparative Analysis, conducted with Chi-square tests (for categorical variables) and *t*-tests (for continuous variables) revealed significant differences between those answering ‘yes’, ‘no’ and ‘I don’t know’ on the dependent variable in respect to region (see Table 2), size, co-placement with other OTs and having an OT in a leading position. Merging with other municipalities emerged as a significant predictor at *P* < .05, while no significant differences were found in respect to the respondent being in a leading position herself. A 2-sided *t*-test suggests that significant differences (*P* < .001) between number of already existing OT-positions show that participants answering ‘no’ had fewer colleagues (5 on average) than those answering ‘yes’ (10.5) or ‘I do not know’ (14). The difference between those answering ‘yes’ and ‘I don’t know’ did not reach statistical significance on this variable.

**Table 2.** Comparative analysis; new OT-positions.

		Yes (*n* = 96)		No (*n* = 240)		Missing (*n* = 225)		Comparative analysis
		*N*	%	*N*	%	*N*	%	χ^2^ (df/p<)
Region	North	8	8.3	23	9.6	29	12.9	
	Middle	7	7.3	35	14.6	34	15.1	
	West	16	16.7	48	20	43	19.1	
	South	15	15.6	44	18.3	10	4.4	
	East	13	13.5	46	19.2	28	12.4	44,785
	South-East	37	38.5	44	18.3	81	36.0	(10/.001)
Size of municipality	<2.000	29	30.2	130	54.2	48	21.3	
	2.000- 19.999	52	54.2	76	31.7	107	47.6	63,922
	>20.000	15	15.6	34	14.2	70	31.1	(4/.001)
Co-placement with other OTs (% yes)		76	79.2	139	57.9	161	71.6	17.501 (2/.001)
OT in leading position		29	30.2	31	12.9	68	30.2	23.342 (2/.001)
Participant in leading position		6	6.3	18	7.5	12	5.3	n.s.
Merged (% yes)		37	38.5	51	21.3	69	30.7	11.513 (2/.01)
Number of OT’s in same unit (mean, std)		10.57 (10.6)		4.95 (5.34)		13.75 (17.32)		

All in all, participants reporting that no new positions had been established in their municipality were significantly different from participants answering ‘yes’ or ‘I don’t know’ to this question in respect to all included variables (except ‘participant being in a leading position’). On the other hand, the only significant difference between participants answering ‘yes’ and ‘I don’t know’ emerged for size of municipality. Taken together, participants answering ‘yes’ or ‘I don’t know’ regarding new positions were most likely to work in the Region of South-East; had a higher likelihood to be co-placed with other OTs; and were, or had been, in the process of merging than participants answering ‘no’ to this question. Regarding size of the municipality, participants who did not know whether new positions had been established tended to work in bigger (>20.000), participants answering ‘yes’ in medium-sized, and participants answering ‘no’ in small municipalities (<2000).

### Initial correlations

All included contextual variables, except for respondent being in a leading position herself, were significantly correlated with the dependent variable. The number of OT-positions within the same unit emerged as the strongest correlate (β = .301), followed by size of the municipality (β = .282), having an OT as a leader (β = .204) and being co-placed together with other OT services (β = .167). Merging was weakly correlated with new positions (β = .125).

The strongest internal correlation (between independent variables) found in this sample was between size of municipality and number of OT positions (β = .475). Moreover, medium-strong internal correlations were found between size of municipality and co-placement (β = .239) and OT in a leading position (β = .194), as well as between number of OT positions and OT in a leading position (β = .234) and co-placement (β = .177).

### Regression analysis

When size of municipality, number of OT-positions, Co-placement and OT in a leading position were entered into regression analysis at 1 single step, only the number of already existing OT-position emerged as significant covariate (β = .308) during regression analysis (Table 3). The model explained 11.9% of the outcome variance in the first step. After introducing ‘merging’ in the second step, the final model explained 13.2% of the variance. Merging emerged as a weak, but significant (β = .129; *P* < .05), positive factor for the establishment of new OT positions. Introducing ‘merging’ into the equation did not make significant changes to any of the other examined relationships.

**Table 3.** Factors influencing the establishment of new OT-position.

*N* = 336	Factors included	Constant	Beta	*R* ^2^
Step1	Size	1.99	−.065	
	Number of OT positions		.308**	
	Co-placement		.094	
	OT in leading position		.005	
	TOTAL			11.9
Step2	Size	2.024	−.054	
	Number of OT positions		.278**	
	Co-placement		.096	
	OT in leading position		.095	
	Merging		.129*	
	TOTAL			13.2

Level of significance: **P < .001. *P < .05.

The described relationships, with ‘number of already existing OT positions’ and ‘merging’ as significant predictors for the establishment of new OT positions prevailed when all variables were entered simultaneously in 1 single step.

## Discussion

This study aimed to explore factors associated with the establishment of new OT-positions in conjunction with the Coordination Reform in 2012. Our results suggest that already existing OT-positions within the municipality emerged as the most consistent predictors of the establishment of new OT-positions. Contrary to our expectations, merging of municipalities emerged as a weak positive factor for the establishment of new OT-positions. Large regional variations emerged, suggesting that most new positions were established in the Region South-east.

Regional differences might be an expression of differences in OT-coverage that existed before the Coordination Reform. This would suggest that the Region of South-east established more new positions due to fewer pre-existing OT-positions. On the other hand, this interpretation is at odds with the above-presented findings, suggesting that most new positions were established in municipalities already offering OT-services. Another possible explanation for regional differences might be related to different internal organisation of services across the included Regions, which implies more or less emphasis on OT, and the fields-of-practice to which they are linked, such as reablement and welfare technology.^8,20^

However, a large proportion of participants (about 40%) answered that they did not know whether new positions had been established in their municipality. This might imply respondent bias and allows for questioning the reliability of the data: positions might have been established that do not show in the present material, simply because other OTs were not aware of them. This implies that findings should be interpreted with great care. On the other hand, comparative analysis suggests that OTs who could not answer this question were most likely to work in big municipalities (while those answering ‘no’ tended to work in small municipalities). Thus, including those who did not give a definitive answer whether new positions had been established would have even reinforced the described trend indicating that most new positions were established in municipalities that already had OT-services. However, missing cases were excluded from further analysis.

All in all, our findings indicate that municipalities that already had OT services before the implementation of the Coordination Reform, were more likely to establish new positions, and this likelihood increased in line with the amount of already existing OTs within the same unit. Does this imply that the majority of new positions established during this period did not contribute to better geographical coverage of OT-services? According to our findings, one might wonder if they even increased geographical inequality, in terms of OT-coverage across municipalities. On the other hand, differences might occur both in respect to whether the municipality has an OT-position or not, and in respect to number of patients per OT.^9,32^ Our findings might describe a necessary increase in OTs in municipalities where OTs experience high workload. Thus, they still might contribute to level out inequality in access to health services by leveling out the number of patients per OT.^9^

Next, our findings may be interpreted in support of prior research suggesting that more knowledge about OT increases the number of OT-positions within municipalities.^26^ As this is also linked to OTs feeling they can make better use of their competencies in interprofessional teams in municipalities with more OTs, it is reasonable to ask whether municipalities wished to increase the number of OTs because they valued their contributions, and could make better use of OTs’ specialized competencies.^26,31,32^ Moreover, municipalities with more OTs can offer a higher degree of internal specialisation, which, in turn, might increase both the number of OT positions, and help to recruit attractive candidates.^17^ The emerging picture suggests that the establishment of OT-positions could indicate the starting point for an upwards-spiral, through which both availability and quality of OT-services increases through better knowledge, and more relevant tasks for community-based OT-services.^17,34^ This might imply major benefits for the communities-in-question; however, this also implies challenges with health equity if some municipalities are ‘left behind’ in respect to this development.

Last, we expected that municipalities that had been, or were in the process of being, merged with other municipalities, had established fewer new OT positions. Our findings suggest the opposite: merging made a small, but significant positive contribution to the establishment of new OT-positions. This counter-argues worries that merging leads to worse OT-coverage by rising the number-of-patients per occupational therapist. Instead, municipalities in the course of restructuring were more likely to establish new OT-positions. It seems like merging raised awareness and highlighted the need for OT services in the affected municipalities. Moreover, it is possible that bigger municipalities also offer more chances for specialisation and the establishment of specialized positions.^32,34^ This, in turn, might point towards an increase in the quality, not only quantity, of community-based OT-services.^9,31^ However, some insecurity is linked to these findings (see below). The need for more knowledge about community-based OT-services is emphasized.

### Methodological consideration

In this article, we aimed to explore if and how contextual factors influenced the establishment of new OT-positions in municipalities. Data were derived from a survey containing self-reported information. This implies some draw-backs for reliability, which became visible in respect to the proportion of missing answers on the variable of-interest: if almost half of the respondents did not know whether a new position had been established or not, can we draw conclusions on the base of the answers we have gathered? Hence, we included a comparative analysis to describe the missing cases and meet insecurity linked to reliability and respondent bias by actively using these insights to interpret findings. All in all, the picture emerges that ‘missing cases’ worked in the biggest municipalities, suggesting that they would not substantially alter our main finding; that new positions were predominantly established in municipalities with prior OT services.

A potential source of error might lie in the recruited sample: participants in this survey were OTs. Municipalities with no community-based OT-service would thereby not have any possible respondent. One might also wonder whether OTs in newly established positions would participate to a lesser degree because of workload and insecurity linked to their new job. Moreover, the design of the study implies that answers and mechanisms from municipalities with more OTs might gain greater importance in our understanding, as more OTs within the municipality also implies more possible participants from this municipality in this study. To reduce insecurity in respect to this, a comparative analysis including missing cases was conducted, and used actively in the discussion of findings and their implications.

Next, and partly due to the major amount of missing cases, some of the sub-groups included in the analysis were small in numbers, which is always linked to statistical insecurity. It is, therefore, possible that some of the other factors included did not reach significance due to small sample sizes, rather than indicating a lack of influence. For example, size of the municipality emerged as a strong predictor in the correlation analysis but lost its significance in the regression analysis. This might be due to excluding missing cases but might also reflect the weaker statistical power due to small sample sizes (*n* = 96).^35^

Moreover, some insecurity is linked to the temporal order which might contradict direct influences in respect to some measures: The Coordination reform was executed in 2012, merging was implemented from 2014,^36^ and this survey was conducted in 2017. In this context, one might wonder how something happening in 2015 could possibly influence whether new positions were established as a consequence of the reform in 2012. However, as indicated above, municipalities were aware that a merging reform was coming already in 2012 and might have adapted their strategies to this anticipated process. Moreover, we argue that ‘positions established as a consequence of the reform in 2012’ could describe positions that were established somewhen during 2012 to 2017. It might take time to gain an overview over all implications of reforms, and positions might have been established later in response to awareness of new roles and responsibilities which crave new, or more, positions or roles in the municipalities.^31^

Last, the final model explains a mere 13% of all variance in the establishment of new OT-positions. Even if this study provides some insights into contextual matters influencing the establishment of new positions, this suggests that we miss out on some, and maybe even more pronounced, factors that contribute to the establishment of new OT-positions. Factors such as municipal economy, changing populations, or political roadmaps might make major contributions, but were not included in this analysis.^31^

### Implications for practice and research

Differences between countries in the organisation of health services and community health care make it difficult to transfer knowledge from one national context to another. However, some of the above-discussed findings might yield valuable knowledge beyond the Norwegian context, if applied and discussed carefully. For example, the notion that OTs are ‘good ambassadors’ for their profession^17-21^ may partly explain our finding that municipalities with already-existing OT-positions were more likely to establish new OT-positions. It is reasonable to expect that this might be true across countries, and might inform strategies for how to raise awareness about the benefits of and increase the number of community-based OT.

Next, the finding that most new positions are established in medium-sized municipalities might partly be explained by these providing increased possibilities for specialisation and participation in professional development and project work, which are described as desirable positions and activities for OTs.^9,17^ Thus, to ensure geographical justice in access to OT-services and increase the attractiveness of less-central positions, one might put an extra effort into developing opportunities for specialisation and competence-building in regions with low OT-coverage. For example, OTs in small municipalities might be offered working time dedicated to developmental work and competence building; small municipalities might join each other into common competence-building projects; or the National Associations might administer national projects aiming to include OTs in rural positions. This finding might be applied to other health professions as well: health workers have gained increased formal competence during the last decades and might prefer to work in positions that offer opportunities to use and expand this competence.

Last, against expectations, we found a weak positive influence of ‘merging’ two municipalities into one. Merging two municipalities is a radical process with extensive organisational changes. It is therefore interesting that these conditions seemed to favour the establishment of new OT-positions. Gaining more insights into how these different kinds of reform influenced communal practice might yield valuavble knowledge for health service planning and municipal administration: The Coordination reform phrased new goals and responsibilities for municipalities, and the regional reform implied deep-plowing changes in the municipal organisation. Investigating if and how these two approaches influenced, and hopefully improved, municipal health services and practices, and to which degree they facilitated for real organisational change rather than attempts to serve new responsibilities within the established organisational framework might yield valuable insights for policy-makers internationally.

## Conclusion

Considering all the above-discussed issues, our findings suggest that the majority of new OT-positions established in conjunction with the Coordination reform occurred in medium-sized municipalities with already existing OT services. This implies that they might not contribute to better geographical OT-coverage across municipalities. However, they might still contribute to reducing inequality in respect to OT-coverage, as new positions in medium-sized municipalities might contribute to level out number-of-patients per OT, as well as differences in the quality of services. Thus, it remains to be known whether the Coordination Reform contributed to better OT-coverage, or whether the newly established positions contributed to close (or even widen) the gap in health by increasing availability and quality of OT-services in municipalities that already offer community-based OT.
